# Multi-Season Multi-Model GWAS Prioritizes Stable Genomic Loci and Candidate Genes for Six Agronomic Traits in Soybean Under Conditions in Southeastern Kazakhstan

**DOI:** 10.3390/plants15182800

**Published:** 2026-09-12

**Authors:** Alibek Zatybekov, Yuliya Genievskaya, Chao Fang, Zixuan Wang, Svetlana Didorenko, Saule Abugalieva, Yerlan Turuspekov

**Affiliations:** 1 Laboratory of Molecular Genetics, Institute of Plant Biology and Biotechnology, Almaty 050040, Kazakhstan; alibek.zatybekov@gmail.com (A.Z.); julia.genievskaya@gmail.com (Y.G.); absaule17@gmail.com (S.A.); 2 School of Life Science, Guangzhou University, Guangzhou 510006, China; fangchao@gzhu.edu.cn; 3 Department of Bioinformatics, College of Life Sciences, Zhejiang University, Hangzhou 310058, China; zixuan.wang@zju.edu.cn; 4 Department of Oil Crop, Kazakh Research Institute of Agriculture and Plant Growing, Almalybak 040909, Kazakhstan; svetl_did@mail.ru

**Keywords:** genotype-by-growing season interaction, whole-genome resequencing, linkage disequilibrium, quantitative trait loci, molecular breeding

## Abstract

Reliable identification of genomic regions controlling complex agronomic traits across variable growing seasons remains a major challenge in soybean genetics and breeding. Here, a diverse panel of 252 soybean accessions was evaluated over six consecutive growing seasons (2018–2023) for flowering time, maturity, plant height, number of seeds per plant, seed yield per plant, and thousand-seed weight. Whole-genome resequencing and variant filtering yielded 2,019,772 high-quality SNPs, and association signals were evaluated using Inclusive Integrative Input Multiple-locus Random-SNP-effect Mixed Linear Model (IIIVmrMLM), Bayesian-information and Linkage-disequilibrium Iteratively Nested Keyway (BLINK), and Multi-Locus Mixed Model (MLMM) together with linkage disequilibrium (LD)-based locus consolidation. Cross-model prioritization retained 21 high-confidence loci supported by all three GWAS models and distributed across 10 chromosomes. Among the identified loci, 18 overlapped or co-localized with previously reported SoyBase genes and QTLs, whereas three (*q.VER2.13-1*, *q.YP.01-1*, and *q.TSW.15-1*) showed no positional overlap with known genes and QTLs and were therefore considered presumably novel. These three loci were associated with flowering time, yield per plant, and thousand-seed weight, accounting for 1.60%, 2.13%, and 5.53% of phenotypic variation, respectively. Ten loci co-localized with genomic regions containing established soybean regulators, including *E2*, *E3*, *GmDt2*, and *POWR1*, support the biological plausibility of the association results. Integration of genomic position, functional annotation, and tissue-expression evidence prioritized 112 candidate genes across 18 loci. These findings provide a focused set of genomic loci and candidate genes for independent validation and further investigation of the genetic basis of soybean adaptation and yield formation under variable continental growing conditions.

## 1. Introduction

Soybean [*Glycine max* (L.) Merr.] is among the world’s most important grain legumes, providing a major source of plant protein and vegetable oil for food, feed, and numerous industrial applications. Sustaining soybean productivity under increasingly variable climatic conditions has become a major breeding challenge, as annual fluctuations in temperature and precipitation strongly influence plant development, phenology, and grain yield. Consequently, identifying genetic factors that confer stable agronomic performance across growing seasons is a key objective of modern soybean improvement [1,2].

Among the agronomic traits targeted in soybean breeding, flowering time, physiological maturity, plant height, seed number, seed weight, and grain yield are particularly important because they collectively determine environmental adaptation and productivity [2,3,4]. Flowering and maturity regulate the duration of vegetative and reproductive growth and consequently influence biomass accumulation, canopy architecture, assimilate partitioning, and seed filling [5,6,7]. Plant height affects lodging resistance and light interception, whereas seed number per plant and thousand-seed weight represent the principal components determining final grain yield [7,8,9]. Importantly, these traits are developmentally interconnected through complex physiological and genetic networks, and their expression is strongly modulated by environmental conditions. Consequently, improving soybean performance requires a comprehensive understanding of both shared and trait-specific genetic determinants governing phenology, plant architecture, and yield formation [2,4].

The genetic architecture of these complex traits is characterized by the combined effects of numerous loci with varying effect sizes, extensive epistatic interactions, and substantial genotype-by-environment (G × E) interactions. Although several major genes controlling flowering and maturity, including members of the *E*, *J*, *Tof*, and *FT* gene families, have been functionally characterized, they explain only part of the observed phenotypic variation [5,6,10,11]. Most agronomic traits remain highly polygenic, with numerous loci contributing small- to moderate-effect alleles that are often influenced by environmental conditions. Therefore, deciphering the stable genetic architecture underlying these traits remains a major objective in soybean genetics and breeding [3,4,12,13].

The rapid development of high-throughput genotyping technologies and the continuous improvement of soybean genomic resources have established genome-wide association studies (GWAS) as one of the most powerful approaches for dissecting complex quantitative traits. By exploiting historical recombination within diverse germplasm collections, GWAS provides considerably higher mapping resolution than conventional biparental linkage mapping while simultaneously capturing a broader spectrum of naturally occurring allelic variation [3,12,14]. Consequently, GWAS has been widely employed to identify loci associated with flowering time, maturity, plant architecture, seed composition, disease resistance, abiotic stress tolerance, and yield components, substantially advancing our understanding of soybean genetics [2,3,4,13].

Despite these advances, the translation of GWAS discoveries into practical breeding remains constrained by the limited reproducibility of many association signals across environments. Numerous loci identified in single-year or single-environment experiments fail to maintain significant effects under different climatic conditions because phenotypic expression is strongly influenced by environmental variability [15,16]. This limitation is particularly pronounced for yield components, where G × E interactions frequently account for a considerable proportion of total phenotypic variance. Consequently, identifying statistically significant loci alone is insufficient; breeding applications require genomic regions that exhibit stable genetic effects despite annual environmental fluctuations [17,18]. Multi-environment GWAS has therefore emerged as an effective strategy for jointly modeling environmental heterogeneity, increasing statistical power, improving the precision of effect estimation, and facilitating the identification of environmentally stable loci with greater breeding value [15,19].

Methodological developments have further improved GWAS’s ability to resolve complex genetic architectures. Traditional single-locus mixed linear models effectively reduce false-positive associations due to population structure and relatedness but often suffer from limited statistical power due to stringent multiple-testing corrections [14,19,20]. To overcome these limitations, many multilocus approaches have been proposed, including MLMM [19], BLINK [20], and the recently developed IIIVmrMLM framework [21]. By simultaneously modeling multiple quantitative trait nucleotides, these methods substantially improve the detection of loci with small and moderate effects while maintaining appropriate control of false discoveries. In particular, IIIVmrMLM extends conventional multilocus association analysis by explicitly incorporating multiple environments and quantitative trait nucleotide-by-environment interactions, thereby enabling a more comprehensive characterization of loci that are environmentally responsive and constitutively expressed [19,20,21]. Such analytical frameworks are particularly well-suited for long-term field experiments in which repeated phenotypic evaluations over consecutive years allow researchers to distinguish stable genetic effects from transient environmental responses [15,16,21].

Although numerous soybean GWASs have been reported during the past decade, relatively few have combined long-term phenotypic evaluation, multi-environment multilocus association analysis, and systematic biological interpretation of identified loci [3,4,13]. Most previous investigations have emphasized either large association panels or multi-location trials conducted over relatively short experimental periods. In contrast, repeated evaluation of genetically diverse germplasm at a single experimental site across multiple consecutive growing seasons provides a complementary framework for assessing the consistency of genetic effects under interannual environmental variation. Such a design minimizes variation attributable to geographic differences among locations while capturing year-to-year variation in climatic conditions. However, stability inferred from this design is specific to growing seasons at the experimental location and does not demonstrate broad adaptation across geographically distinct environments [15,22,23].

This strategy is particularly vital for soybean production in continental climates. Although previous GWASs have provided valuable insights into soybean genetics, identifying stably expressed genomic regions across highly fluctuating seasons remains a challenge [2,24,25,26,27,28,29]. In Kazakhstan, soybean production is centered in the southeastern region, where pronounced year-to-year variation in temperature, precipitation, and season length strongly impacts crop performance. In this environment, flowering and maturity dictate the synchronization of growth with favorable environmental conditions, whereas plant height and yield components are heavily shaped by seasonal shifts. Therefore, leveraging multi-year GWAS to complement existing genetic mapping data is crucial for discovering resilient genomic regions that can drive the development of climate-adapted soybean cultivars.

Equally important is the biological interpretation of statistically significant association signals. Advances in soybean reference genomes, functional annotation, transcriptomics, and curated genomic resources such as SoyBase have substantially enhanced the identification of biologically plausible candidate genes underlying GWAS loci [30,31,32]. Integrating association mapping with linkage disequilibrium information, previously reported quantitative trait loci, comparative functional annotation, and gene ontology analyses provides stronger evidence for prioritizing candidate genes and facilitates the transition from statistical associations to biological mechanisms and molecular breeding applications [3,13,33,34].

Therefore, the objectives of this study were to (i) characterize phenotypic variation, variance components, and broad-sense heritability for six major agronomic traits in a globally diverse soybean panel evaluated over six consecutive growing seasons; (ii) identify stable genomic loci underlying phenological, morphological, and yield-related traits by integrating long-term phenotyping, best linear unbiased estimators (BLUE)-based phenotypic adjustment, whole-genome resequencing, and complementary GWAS approaches; (iii) prioritize robust genomic regions through linkage disequilibrium-based locus consolidation and independent cross-model validation; (iv) identify and functionally characterize candidate genes underlying the prioritized stable loci using genomic annotation, Gene Ontology classification, and tissue-specific expression analyses; and (v) evaluate the potential application of the identified stable genomic loci and candidate genes as genomic resources for molecular breeding and soybean improvement under continental environmental conditions.

## 2. Results

### 2.1. Phenotypic Variation Across Six Growing Seasons

**Figure 1:**
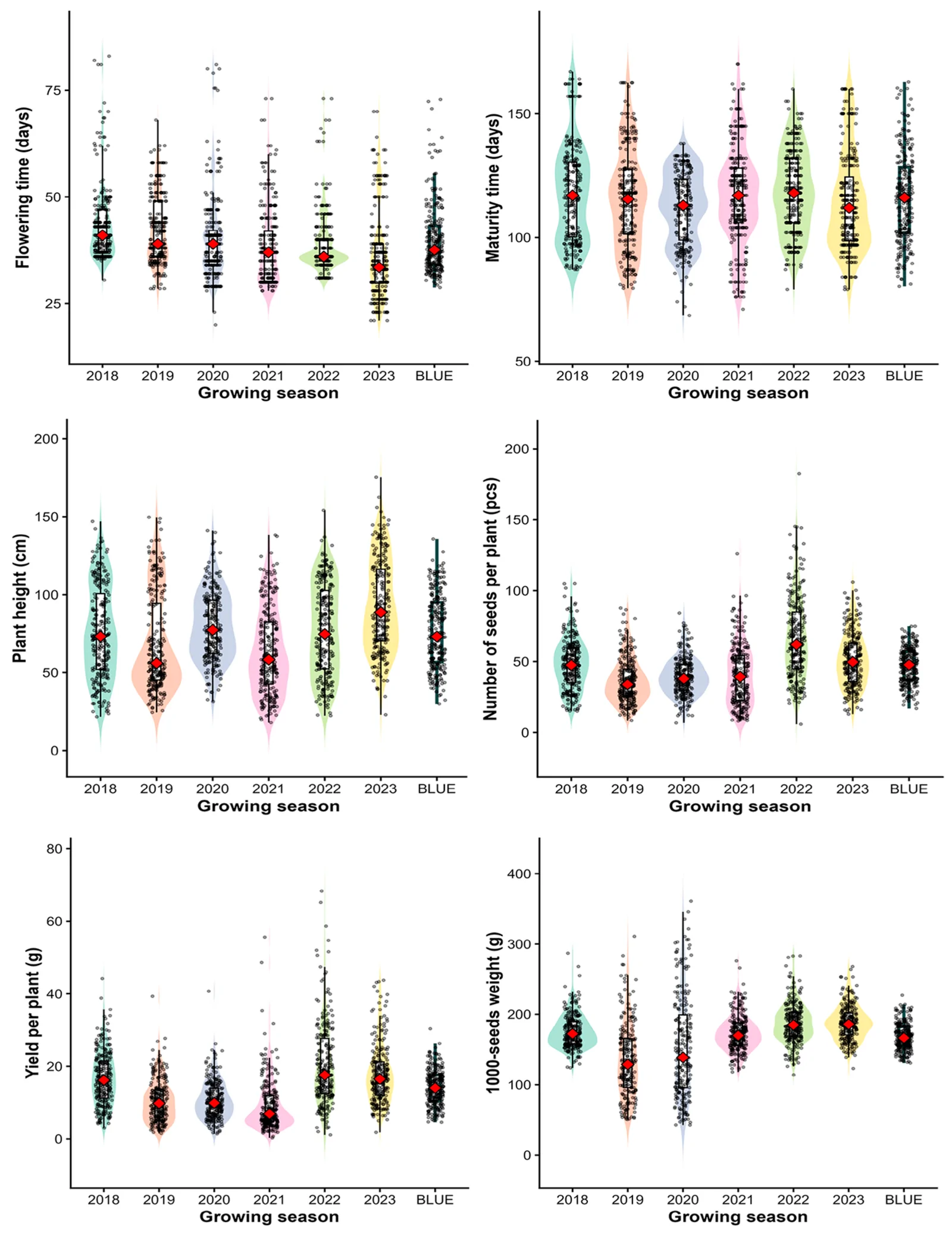
Phenotypic variation in soybean agronomic traits across six growing seasons (2018–2023) and their BLUE values.

Considerable phenotypic variation was observed for all six agronomic traits, with trait-dependent differences in the magnitude of interannual variation across the six growing seasons. All traits exhibited continuous phenotypic distributions characteristic of quantitative inheritance, although their distributions and descriptive statistics differed among years (Figure 1 and Table S1). The broad phenotypic ranges observed for each trait indicated sufficient phenotypic variation for subsequent GWAS.

Phenological traits exhibited relatively consistent variation across growing seasons. The mean of flowering time (VER2) gradually decreased from 43.6 days in 2018 to 36.0 days in 2023, whereas the mean of maturity time (VER8) ranged from 112 to 118 days. Both traits exhibited lower coefficients of variation (CV) than the yield-related traits. Nevertheless, differences among growing seasons were observed for both VER2 and VER8, reflecting seasonal variation in phenological development.

Greater seasonal variation was observed for plant height (PH) and the yield-related traits. PH, number of seeds per plant (NSP), and yield per plant (YP) exhibited broad phenotypic ranges and high CV values across growing seasons. Among these traits, YP showed the greatest interannual variation, whereas thousand-seed weight (TSW) exhibited comparatively smaller seasonal fluctuations (Figure 1 and Table S1).

The BLUE values calculated across six growing seasons showed intermediate distributions relative to the individual growing-season observations (Figure 1). Compared with single-year data, BLUE distributions were generally narrower for PH, NSP, and YP, whereas those for VER2, VER8, and TSW remained largely comparable to those observed within individual growing seasons. The BLUE estimates were subsequently used as the primary phenotypic dataset for GWAS.

**Figure 2:**
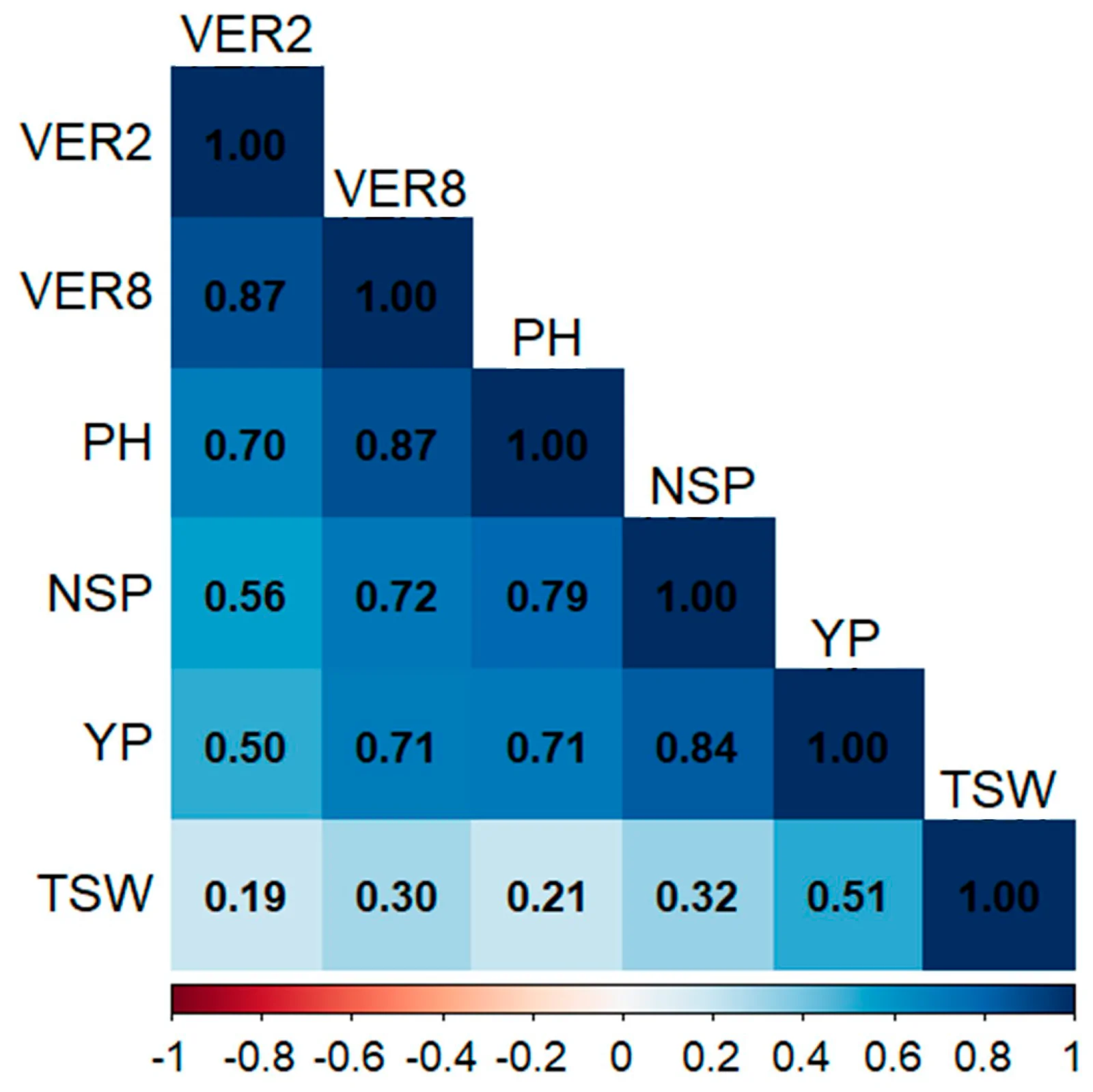
Pearson correlation matrix among six agronomic traits calculated using BLUE values across six growing seasons (*p* < 0.05). The flowering time (VER2), maturity time (VER8), plant height (PH), number of seeds per plant (NSP), yield per plant (YP), and thousand-seed weight (TSW).

Pearson correlation analysis based on the BLUE values revealed strong positive correlations among agronomic traits (Figure 2).

The strongest correlations were observed between VER2 and VER8 (*r* = 0.87), followed by VER8 and PH (*r* = 0.87). Among the yield-related traits, NSP showed the strongest positive correlation with YP (*r* = 0.84). PH was also positively correlated with NSP (*r* = 0.79) and YP (*r* = 0.71), whereas TSW exhibited only weak to moderate correlations with the remaining traits.

### 2.2. ANOVA and Heritability

Analysis of variance (ANOVA) showed significant effects of genotype, growing season, and genotype × growing season interaction on all six evaluated traits (Table 1).

*H*^2^ ranged from 0.235 to 0.913, with the highest estimates for VER8 (0.913), PH (0.758), and VER2 (0.743), and lower for NSP (0.348), YP (0.313), and TSW (0.235). Genotypic effects were significant for all traits (*p* < 2 × 10^−16^), with *F*-values ranging from 14.64 for NSP to 427.98 for VER8, while growing season effects ranged from *F* = 201.88 for VER8 to *F* = 1649.33 for VER2. Significant genotype × growing season effects were also detected for all traits, with F-values ranging from 2.60 for PH to 55.83 for TSW. Overall, *H*^2^ estimates decreased in the order VER8 > PH > VER2 > NSP > YP > TSW (Table 1).

### 2.3. Genotyping Data and Linkage Disequilibrium

**Figure 3:**
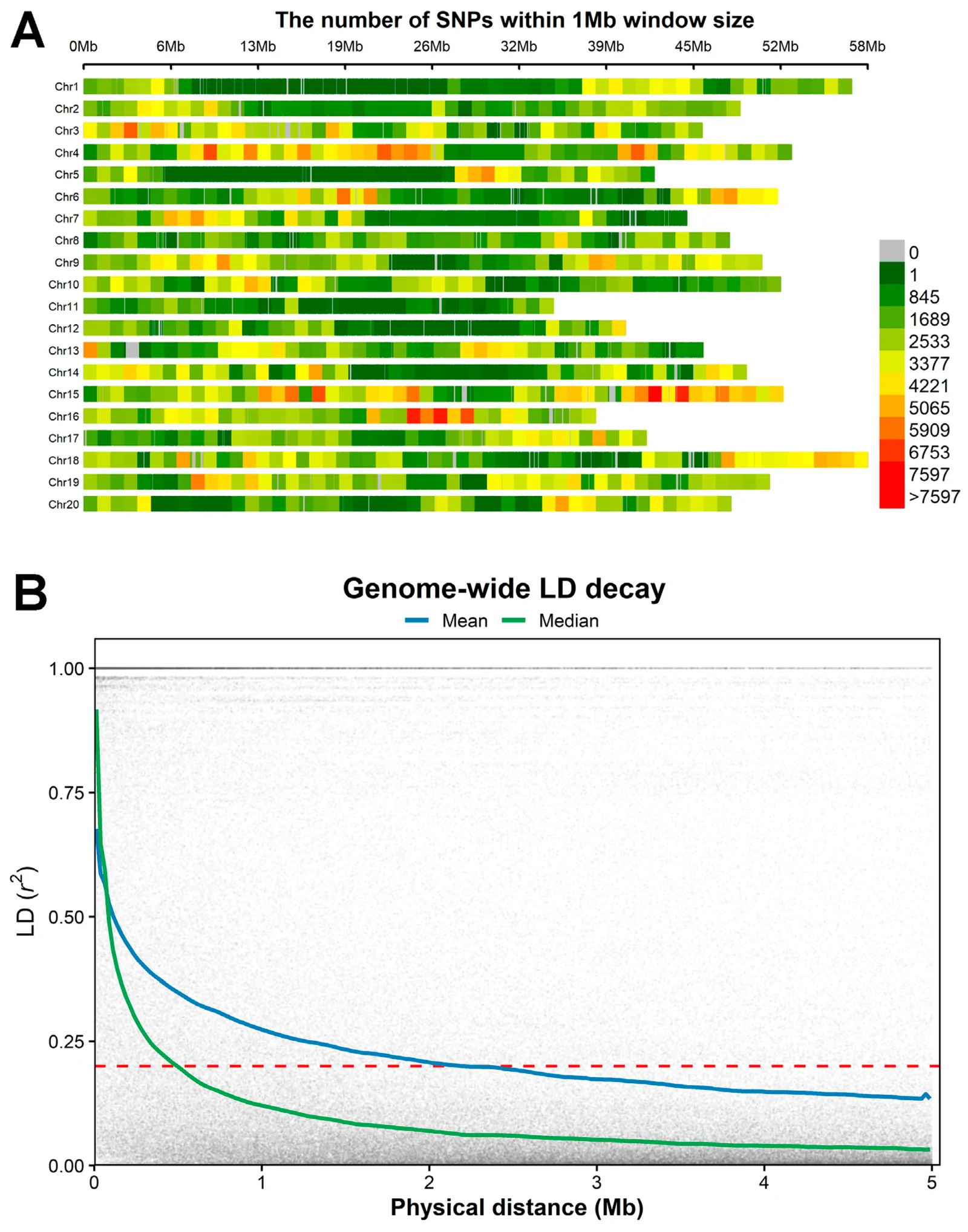
Genome-wide marker density and linkage disequilibrium decay in the soybean association panel. (**A**) Distribution of 2,019,772 high-quality SNP markers across the 20 soybean chromosomes, calculated using consecutive 1 Mb genomic windows. (**B**) Genome-wide linkage disequilibrium decay estimated from pairwise intrachromosomal SNP comparisons. Grey dots represent sampled pairwise LD (*r*^2^) values, whereas the blue and green curves correspond to the five-point moving-average smoothed mean and median LD estimates, respectively. The dashed red line indicates the LD threshold (*r*^2^ = 0.20) used to estimate genome-wide LD decay.

High-quality SNP markers were densely distributed across all 20 soybean chromosomes, providing nearly complete genome-wide marker coverage for association mapping (Figure 3A).

Genome-wide LD analysis revealed a progressive decline in pairwise LD with increasing physical distance between SNP markers (Figure 3B). High LD values were observed among closely linked markers, followed by a gradual decrease toward background levels with increasing physical distance. Pairwise LD estimates were summarized across consecutive 25 kb physical distance bins, and the resulting decay curve was smoothed using a five-point moving average to reduce stochastic fluctuations in pairwise LD estimates. Based on the smoothed genome-wide mean LD curve, the LD decay threshold (*r*^2^ = 0.20) was reached at 2.196 Mb (Figure 3B). Similar LD decay patterns were observed across individual chromosomes, although the extent of LD varied among chromosomes (Figure S1).

### 2.4. Population Structure

**Figure 4:**
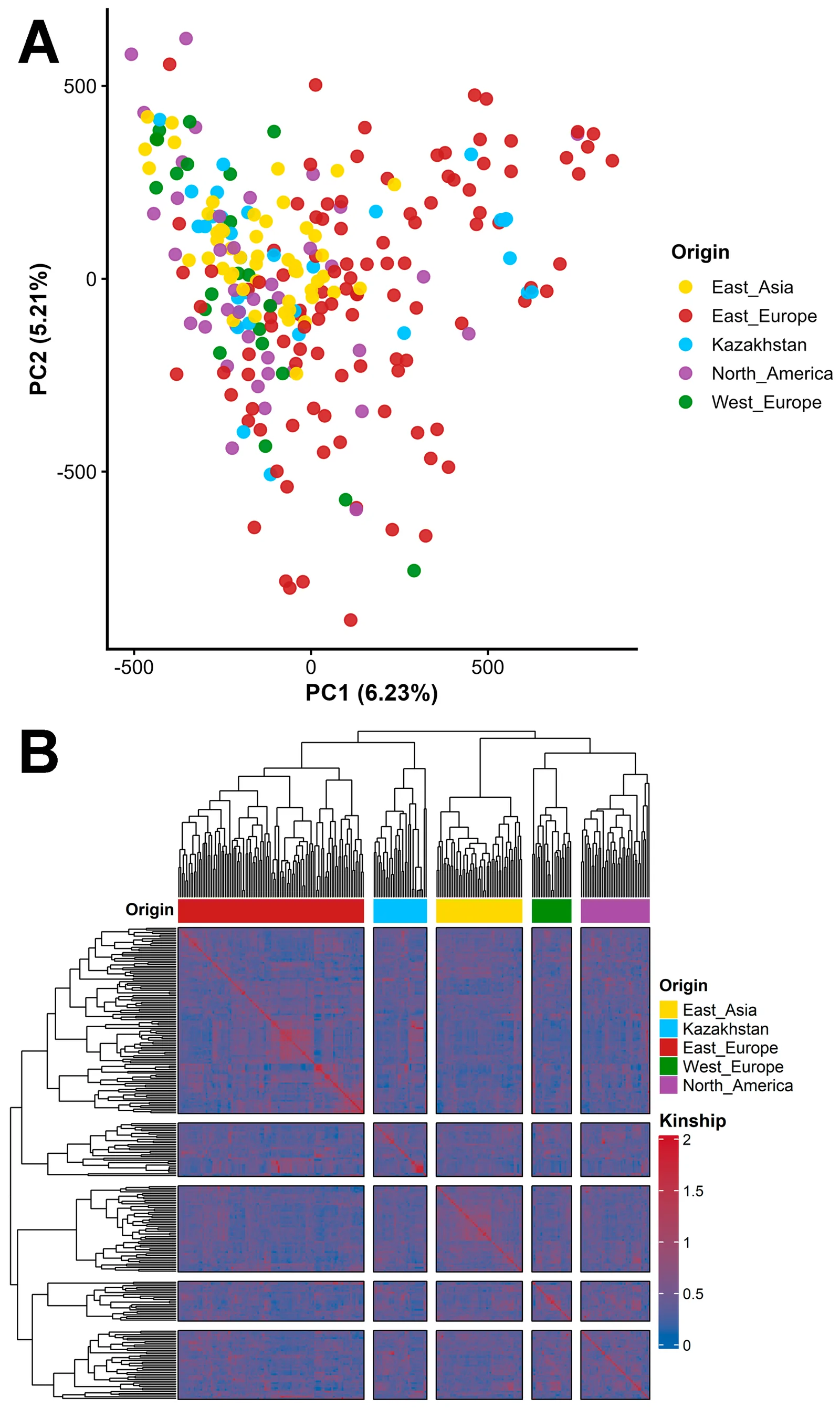
Population genetic structure of the soybean association panel. (**A**) Principal component analysis (PCA) based on genome-wide SNP markers. (**B**) Genomic kinship heatmap illustrating pairwise genetic relatedness among the 252 soybean accessions.

PCA and the genomic relationship matrix revealed moderate population structure within the soybean association panel (Figure 4).

The first two principal components explained 11.44% of the total molecular variation, indicating relatively weak population stratification despite the broad geographic origin of the accessions (Figure 4A). Accessions from similar geographic regions tended to form partially overlapping clusters, with substantial overlap among regional groups in the PCA plot.

The genomic kinship matrix supported the PCA results by showing generally weak pairwise genetic relationships among the 252 accessions, with only a limited number of closely related genotypes (Figure 4B). These results indicate that the association panel captured broad genetic diversity while exhibiting only moderate population structure.

### 2.5. Genome-Wide Association Analysis and Prioritization of Robust Association Loci

Across the six evaluated agronomic traits, initial multi-locus GWAS screening retained 8537 marker–trait associations (MTAs) at *p* < 1.0 × 10^−4^. Among these detected signals, 124 MTAs were identified by IIIVmrMLM, 6679 by BLINK, and 1734 by MLMM (Table S2). To reduce redundancy arising from clusters of highly linked SNPs, associations retained at the initial screening stage were subsequently consolidated into genomic loci according to the genome-wide LD structure. SNPs within the same LD-defined interval (2.196 Mb) were grouped into a single QTL, represented by the lead SNP with the strongest statistical support. This procedure reduced the number of association signals from 8537 individual SNPs to 198 non-redundant genomic loci. Among these 198 loci, 118 were detected by only a single GWAS framework (including 51 identified by IIIVmrMLM, 47 by BLINK, and 20 by MLMM), whereas the remaining 80 were detected by two or more models (Table S2).

The robustness of the identified loci was subsequently evaluated by comparing the results obtained with IIIVmrMLM, BLINK, and MLMM. A total of 80 loci were consistently supported by at least two GWAS models and were retained as validated loci for subsequent analyses. The number of validated loci differed among agronomic traits. VER2 exhibited the largest number of validated loci (18), followed by PH (17), NSP (14), VER8 (13), TSW (11), and YP (7) (Table S2). These 80 cross-model-validated loci provided the basis for subsequently identifying of the most robust association signals. Application of the more stringent criterion requiring support from all three GWAS models further reduced this set to 21 high-confidence stable loci, which were retained for comparison with previously reported QTLs and candidate-gene analyses.

### 2.6. High-Confidence Stable Genomic Loci and Their Relationship with Known Soybean Genes and QTLs

Application of the stringent cross-model concordance criterion identified 21 high-confidence stable loci associated with the six agronomic traits. These loci were distributed across 10 soybean chromosomes and were supported by all three GWAS models (Table 2).

The largest numbers of high-confidence stable loci were identified for VER2 (5) and PH (5), followed by NSP (4), VER8 (3), TSW (2), and YP (2). These 21 loci were retained because they were concordantly supported by IIIVmrMLM, BLINK, and MLMM after LD-based locus consolidation (Table 2). The Bonferroni-corrected genome-wide significance threshold based on 2,019,772 SNPs was *p* < 2.48 × 10^−8^. Eighteen lead associations exceeded this stringent threshold, whereas the remaining three were retained based on cross-model concordance despite not individually reaching Bonferroni-corrected significance. Accordingly, cross-model support and Bonferroni-corrected genome-wide significance are treated as distinct criteria in the present study.

**Figure 5:**
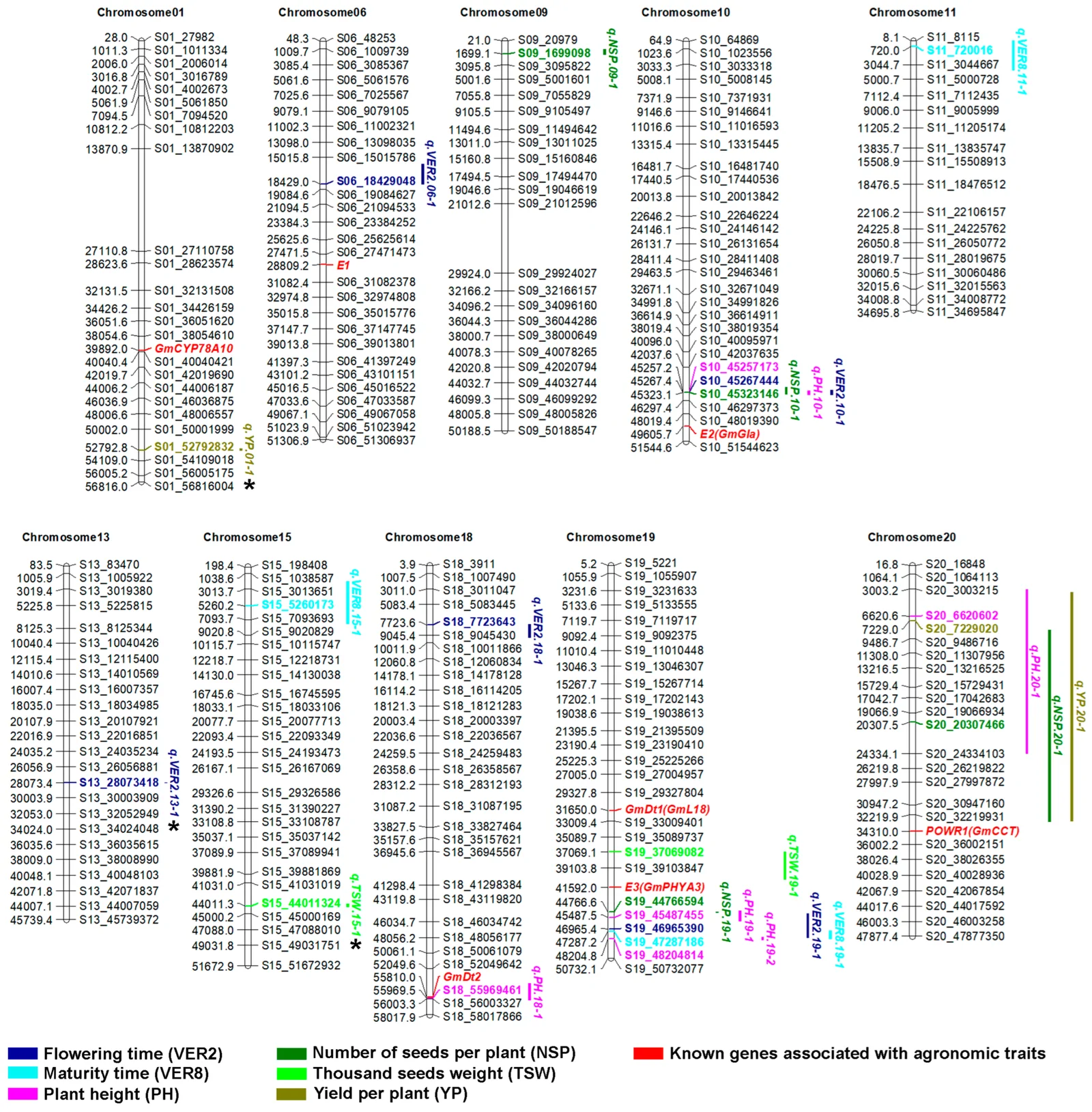
Genomic distribution of the high-confidence stable loci identified by multi-season GWAS and their physical proximity to previously characterized soybean genes. SNP positions are shown in kb to the left of each chromosome. *—presumably novel QTLs based on the absence of positional overlap with previously reported SoyBase QTLs.

Comparison of the physical positions of the high-confidence stable loci with previously characterized soybean genes revealed 10 cases in which the identified loci were located in close physical proximity to major genes controlling phenological, morphological, and yield-related traits (Figure 5).

Chromosome 19 contained the largest number of co-localized loci. On chromosome 10, *q.VER2.10-1*, *q.NSP.10-1* and *q.PH.10-1* co-localized with the *E2* (*GmGIa*) region. Multiple loci on chromosome 19 (*q.VER2.19-1*, *q.PH.19-1*, *q.NSP.19-1*, and *q.TSW.19-1*) co-localized with the genomic interval containing *E3* (*GmPhyA3*). Additional co-localization was observed between *q.PH.18-1* and *GmDt2*, whereas *q.NSP.20-1* and *q.YP.20-1* mapped within the genomic region harboring *POWR1/GmCCT* (Table 3).

The 21 high-confidence stable QTLs identified in this study were compared with previously reported soybean QTLs available in the SoyBase database (Table 3 and Table S3). Of the 21 high-confidence stable loci, 17 showed positional overlap with at least one previously reported SoyBase QTL, whereas no overlapping QTLs were detected for *q.VER2.13-1*, *q.NSP.19-1*, *q.YP.01-1*, and *q.TSW.15-1*. These four loci were associated with distinct components of soybean adaptation and productivity: VER2 (*q.VER2.13-1*), NSP (*q.NSP.19-1*), YP (*q.YP.01-1*), and TSW (*q.TSW.15-1*). Their lead SNPs explained 1.60%, 4.36%, 2.13%, and 5.53% of the phenotypic variation, respectively (Table 2).

The number of overlapping SoyBase QTLs ranged from one to 66. The highest numbers of overlaps were recorded for *q.VER2.19-1* (66), *q.VER8.15-1* (46), *q.VER2.06-1* and *q.YP.20-1* (26), and *q.NSP.20-1* (22). The overlapping SoyBase QTLs were assigned mainly to the seed composition and yield, plant architecture, and disease and stress categories, with six QTLs classified in the “other” category. The previously reported traits within the overlapping regions included first flowering, reproductive period, R8 full maturity, plant height, node number, stem termination type, canopy width, interbranch length, pod number, seed set, seed weight, seed yield, seed protein and oil content, as well as disease- and stress-related traits. The complete list of overlapping SoyBase QTLs, including their categories, QTL types, names, and physical positions, is provided in Table S3.

### 2.7. Candidate Gene Identification, Functional Annotation, and Expression Profiling

Candidate genes were identified within the LD intervals surrounding the 21 high-confidence stable loci using the Zhonghuang 13 (ZH13 v2.0) reference genome annotation (Table S4).

**Figure 6:**
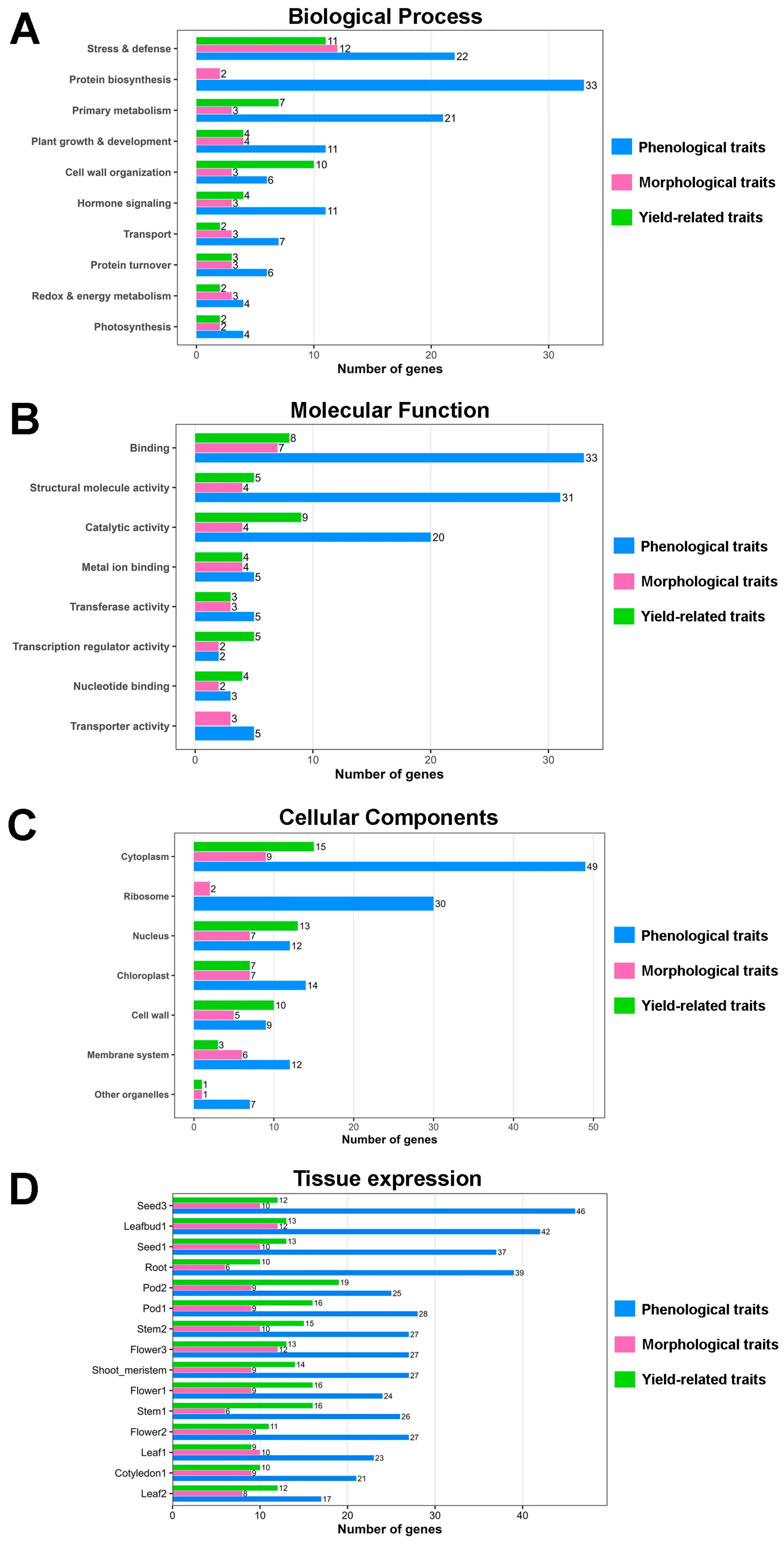
Functional classification and tissue-specific expression profiles of candidate genes identified within the high-confidence genomic loci. (**A**–**C**) GO-based functional classification of candidate genes according to Biological Process (**A**), Molecular Function (**B**), and Cellular Component (**C**). Related GO annotations were consolidated into broader functional categories for visualization. (**D**) Tissue-specific expression profiles of candidate genes across major soybean organs and developmental stages based on the SoyMD transcriptome dataset.

Gene Ontology (GO) analysis revealed a broad distribution of candidate genes across biological process categories, with clear differences among phenological, morphological, and yield-related traits (Figure 6A). Candidate genes associated with phenological traits predominated in most categories, particularly protein biosynthesis (33 genes), stress and defense (22), and primary metabolism (21), followed by plant growth and development (11), hormone signaling (11), transport (7), cell wall organization and protein turnover (6 each), and redox and energy metabolism and photosynthesis (4 each). For morphological traits, the largest group was associated with stress and defense (12 genes), whereas the remaining categories contained two to four genes each. Candidate genes related to yield-related traits were most frequently assigned to stress and defense (11 genes) and cell wall organization (10), followed by primary metabolism (7), plant growth and development (4), hormone signaling and protein turnover (3 each), and transport, redox and energy metabolism, and photosynthesis (2 each). Overall, 125, 39, and 44 gene-category assignments were recorded for phenological, morphological, and yield-related traits, respectively.

Molecular function analysis showed a similar predominance of candidate genes associated with phenological traits (Figure 6B). Among these genes, binding (33 genes) and structural molecule activity (31) were the largest functional groups, followed by catalytic activity (20), metal ion binding, transferase activity, and transporter activity (5 each), nucleotide binding (3), and transcription regulator activity (2). For morphological traits, binding was the largest category (7 genes), followed by structural molecule activity, catalytic activity, and metal ion binding (4 genes each), transferase and transporter activities (3 genes each), and transcription regulator activity and nucleotide binding (2 genes each). Among genes associated with yield-related traits, catalytic activity (9 genes) and binding (8 genes) were the most frequent molecular functions, followed by structural molecule activity and transcription regulator activity (5 each), metal ion binding and nucleotide binding (4 each), and transferase activity (3). In total, 104, 29, and 38 molecular-function assignments were recorded for phenological, morphological, and yield-related traits, respectively.

Cellular component analysis showed that the cytoplasm contained the largest number of candidate proteins across all three trait groups (Figure 6C), comprising 49 genes associated with phenological traits, 9 with morphological traits, and 15 with yield-related traits. Among phenology-related genes, the cytoplasm was followed by the ribosome (30 genes), chloroplast (14), nucleus and membrane system (12 each), cell wall (9), and other organelles (7). For morphological traits, genes were distributed among the cytoplasm (9), nucleus (7), chloroplast (7), membrane system (6), cell wall (5), ribosome (2), and other organelles (1). Yield-related genes were most frequently assigned to the cytoplasm (15), nucleus (13), and cell wall (10), followed by the chloroplast (7), membrane system (3), and other organelles (1). Overall, the cellular-component categories contained 133, 37, and 49 assignments for phenological, morphological, and yield-related traits, respectively.

Tissue-specific expression profiling further revealed differences in the distribution of highly expressed candidate genes among the three trait groups (Figure 6D). Phenology-related genes were the most abundant group across all 15 tissues examined, with the highest numbers detected in Seed3 (46 genes), Leafbud1 (42), Root (39), and Seed1 (37). Intermediate numbers were observed in Pod1 (28), Stem2, Flower3, Shoot_meristem, and Flower2 (27 each), Stem1 (26), Pod2 (25), Flower1 (24), Leaf1 (23), and Cotyledon1 (21), whereas Leaf2 contained 17 genes. Morphological-trait genes ranged from 6 to 12 across tissues, with the highest number in Leafbud1 (12), followed by Flower3 (11), and Seed3, Seed1, Stem2, and Cotyledon1 (10 each). Yield-related genes ranged from 10 to 19, with the highest numbers observed in Pod2 (19), Pod1, Flower1, and Stem1 (16 each), Stem2 (15), and Shoot_meristem (14). Across all tissues, 436, 136, and 200 gene-tissue occurrences were recorded for phenological, morphological, and yield-related traits, respectively.

**Figure 7:**
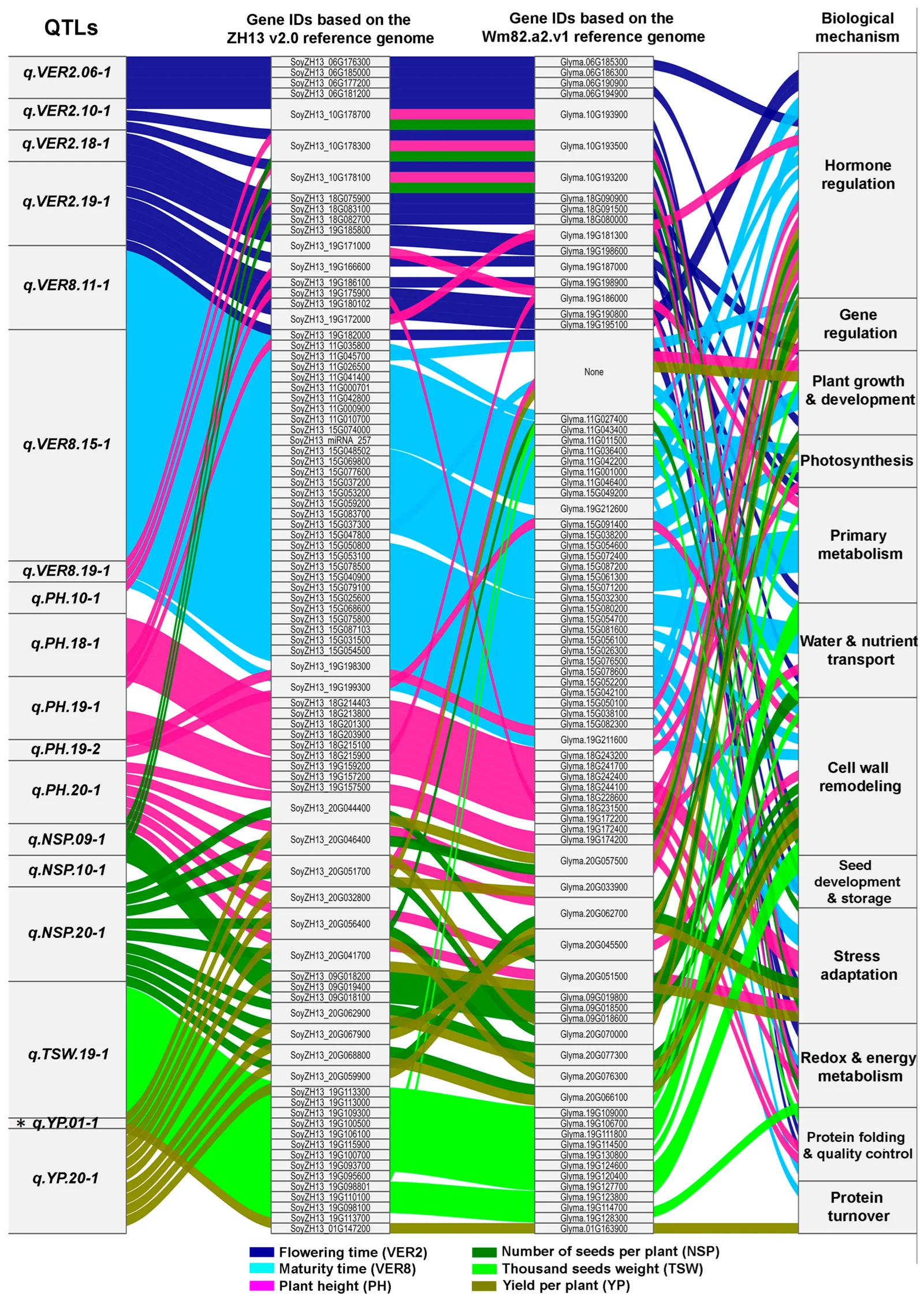
Integrative functional classification of candidate genes within 18 high-confidence stable genomic loci associated with phenological, morphological, and yield-related traits in soybean and their predicted biological mechanisms. Candidate gene IDs are shown according to the ZH13 v2.0 reference genome, along with their corresponding IDs in the Wm82.a2.v1 reference genome. “None” indicates that no corresponding gene ID was identified in the Wm82.a2.v1 genome annotation for the respective ZH13 v2.0 candidate gene. *—presumably novel QTLs based on the absence of positional overlap with previously reported SoyBase QTLs.

Among the 21 identified stable QTLs, 18 contained candidate genes exhibiting high transcript abundance with expression levels exceeding 200 TPM. Across these 18 high-confidence stable loci, 112 candidate genes were prioritized based on their functional annotations, expression evidence, and putative biological relevance to the investigated traits and associated biological processes (Figure 7 and Table S5).

These 112 prioritized genes were classified into 12 major biological mechanisms. The largest functional categories were involved in hormone regulation (18 genes), cell wall remodeling (15 genes), primary metabolism (12 genes), and stress and phenology (11 genes). Gene regulation, as well as water and nutrient transport, comprised nine genes each, followed by redox and energy metabolism (8 genes) and plant growth and development (8 genes). The remaining genes were assigned to protein folding and quality control (7 genes), protein turnover (5 genes), photosynthesis (5 genes), and seed development and storage (5 genes).

The integrated analysis prioritized candidate genes by combining their genomic positions within the identified loci, functional annotations, and evidence of tissue-specific expression. Genes were retained based on the convergence of these complementary criteria and their putative biological relevance to the investigated traits or associated biological processes. Figure 6 summarizes the functional classification and tissue-specific expression patterns of the candidate genes, whereas Figure 7 provides an integrative overview linking the 112 prioritized genes to their corresponding genomic loci and putative biological mechanisms. Detailed gene-level functional annotations and expression profiles are provided in Table S4, while Table S5 summarizes the prioritized genes, their genomic positions in the ZH13 v2.0 and Wm82.a2.v1 reference genomes, gene symbols, functional descriptions, and assigned biological mechanisms. Together, these complementary datasets provide the rationale for candidate prioritization but should not be interpreted as evidence of causality.

## 3. Discussion

### 3.1. Multi-Season Phenotyping and BLUE Estimation Provide a Robust Phenotypic Foundation for GWAS

Reliable GWAS mapping depends on the accuracy and environmental stability of phenotypic estimates, as phenotypic uncertainty can substantially reduce the reproducibility of marker–trait associations. In this study, phenotypic evaluation across six growing seasons captured interannual environmental variation while maintaining a consistent experimental site and management conditions (Figure 1 and Table S1). Genotype-specific phenotypic values across seasons were estimated using BLUEs, with genotype treated as a fixed effect, thereby accounting for environmental variation while retaining differences among accessions for subsequent GWAS [22,23]. The importance of multi-season phenotypic evaluation and mixed-model approaches for improving the precision of phenotypic estimates and downstream genetic analyses has been well established in quantitative genetic studies [15,22,23].

ANOVA revealed contrasting patterns of genetic and environmental control among the investigated traits. VER2, VER8, and PH exhibited high *H*^2^ values and comparatively large genotypic contributions, indicating relatively stable genetic control across growing seasons (Table 1). In contrast, NSP, YP, and, particularly, TSW showed greater contributions from the growing season and the genotype × growing season interaction, indicating stronger environmental dependence of their phenotypic variation [2,27]. This distinction between developmental and productivity-related traits is consistent with previous soybean studies showing stronger genetic control of developmental traits and greater environmental and genotype × growing season effects on yield-related traits [3,4].

Notably, TSW showed the lowest across-season broad-sense heritability (*H*^2^ = 0.235) despite its comparatively narrower phenotypic fluctuations, while exhibiting the strongest genotype × growing season interaction among the evaluated traits (F = 55.83) (Table 1). This result indicates substantial season-dependent variation in seed weight expression across the six growing seasons and may partly explain why only a limited number of stable TSW loci were retained after the complete GWAS prioritization procedure [35,36]. The use of across-season BLUEs was consistent with the primary objective of identifying genomic associations with season-adjusted genotype performance across the six-year experiment. However, this approach can attenuate genetic effects that are strongly dependent on individual growing seasons and therefore does not capture the complete spectrum of genotype × growing season-specific associations. Consequently, the loci retained in the present analysis should be interpreted primarily as robust associations with across-season genotype performance, whereas complementary year-specific or explicit multi-environment GWAS using growing season-specific phenotypes would be required to characterize environmentally responsive loci in greater detail.

The association panel exhibited broad geographic diversity, moderate population structure, and weak pairwise relatedness. Together with 2,019,772 high-quality SNPs and genome-wide LD decay of 2.196 Mb (Figure 3), these characteristics provided a suitable genomic framework for association mapping and LD-based locus definition. Broad genetic diversity, appropriate control of population structure, and dense genome-wide marker coverage are important factors for reliable association mapping in soybean [12,14]. Thus, the combination of six-year phenotypic evaluation, mixed-model phenotypic estimation, characterization of genotype × growing season effects, and high-density genomic data provided the framework for the multi-model GWAS analysis described below.

Importantly, because all six growing seasons were evaluated at the same experimental site, the present study should be regarded as a multi-season, single-location experiment rather than a multi-location environmental trial. Consequently, the stability inferred here refers to the consistency of genetic effects across interannual environmental variation at this location. Although this design provides evidence for temporal reproducibility of the identified associations, validation across geographically distinct environments will be required before broader environmental stability can be established.

### 3.2. Integrated GWAS Strategy for High-Confidence Stable Loci Identification

One of the major challenges in GWAS is distinguishing biologically meaningful associations from redundant or model-specific signals. High-density genomic datasets often contain numerous significant SNPs that are correlated within the same LD block, making post-GWAS prioritization essential for identifying reproducible loci with biological and breeding relevance [19,20,37]. In this study, significant associations identified by the multi-season IIIVmrMLM analysis were first consolidated into genomic loci based on the observed LD structure and subsequently evaluated using the complementary BLINK and MLMM approaches.

The hierarchical filtering procedure markedly reduced the initial association space. LD-based consolidation reduced 8537 significant marker–trait associations to 198 non-redundant genomic loci, representing a 97.7% reduction in redundant association signals. Cross-model comparison subsequently retained 80 loci supported by at least two GWAS models, representing 40.4% of the LD-defined loci (Table S2). Applying the more stringent requirement for concordant support across all three GWAS models further reduced this set to 21 high-confidence stable loci (Table 2). Thus, only 0.25% of the initial marker–trait associations ultimately contributed to the final set of high-confidence stable loci. This progressive reduction illustrates the stringency of the combined LD-based consolidation, cross-model validation, and stability-based prioritization framework and substantially narrows the genomic regions requiring subsequent biological interpretation [4,37].

The three GWAS approaches provided complementary statistical frameworks for evaluating the same across-season BLUE phenotypes. IIIVmrMLM, BLINK, and MLMM differ in their procedures for identifying and controlling multiple associated loci [19,20,21], and concordant detection across these approaches was therefore used as an additional criterion for prioritizing robust associations. Importantly, although IIIVmrMLM can model QTN-by-environment interactions, all three GWAS approaches in the present study were applied to genotype-specific BLUEs estimated across the six growing seasons. Consequently, QTN × growing season interactions were not directly modeled in the GWAS, and the resulting loci represent associations with season-adjusted genotype performance rather than growing season-specific marker effects.

### 3.3. Stable Genetic Regulation of Studied Agronomic Traits

The recovery of genomic regions in physical proximity to well-characterized soybean developmental genes provides biological support for the association-mapping framework used in the present study. Seven loci associated with flowering and maturity were located near the major maturity genes *E2* (*GmGIa*) and *E3* (*GmPhyA3*), which are key components of the photoperiod-response network regulating soybean flowering and maturity [5,6,7]. In particular, the strong associations detected in the *E3*-containing chromosome regions are consistent with the major contribution of photoperiod-response pathways to phenological variation in soybean (Figure 5).

The identified loci also showed positional correspondence with major regulators of plant architecture and productivity. The PH-associated *q.PH.18-1* was located near *GmDt2*, a major regulator of stem growth habit and plant architecture [38], whereas loci associated with NSP and YP on chromosome 20 occurred in the broader genomic region containing *POWR1/GmCCT*, which has been implicated in the coordinated regulation of soybean seed composition and productivity [9] (Figure 5). Together, the recovery of genomic regions associated with established developmental regulators supports the biological relevance of the high-confidence loci identified by the multi-model GWAS approach.

### 3.4. Presumably Novel Stable Genomic Regions Associated with Phenology and Yield Components

The most distinctive outcome of the present analysis was the identification of three high-confidence loci (*q.VER2.13-1*, *q.YP.01-1*, and *q.TSW.15-1*) that showed no positional overlap with genes and QTLs included in our SoyBase comparison (Table 3) [30]. We therefore refer to these loci as presumably novel rather than definitively novel, because the absence of overlap in the database comparison does not exclude all previously reported associations within the corresponding genomic regions.

*q.VER2.13-1* was associated with flowering time and explained 1.60% of phenotypic variation. Unlike two other phenology-associated loci recovered near established maturity-gene regions, this chromosome 13 interval showed no overlap with the SoyBase QTLs included in our comparison, suggesting an additional moderate-effect region contributing to VER2 variation in this panel [3,4].

The remaining two presumably novel loci were associated with yield components that showed comparatively low across-season heritability and significant genotype × growing season effects [21]. Nevertheless, *q.YP.01-1* and *q.TSW.15-1* were supported by all three GWAS models and explained 2.13% and 5.53% of phenotypic variation, respectively (Table 3). In particular, *q.TSW.15-1* represent promising targets for independent validation because of their comparatively larger estimated effects.

These three regions should therefore be considered candidate genomic resources rather than immediately deployable breeding loci. Independent validation across populations and environments, followed by haplotype analysis, higher-resolution mapping, and functional characterization, will be required to establish their reproducibility, identify favorable alleles, and estimate their potential breeding value [37,38,39].

### 3.5. Integrative Prioritization of Candidate Genes Strengthens the Biological Interpretation of Stable Genomic Loci

Identifying statistically robust GWAS loci is an important step toward resolving the genetic basis of complex agronomic traits. However, LD-defined intervals may span multiple genes, making it challenging to identify causal genes from association signals alone. This limitation is particularly relevant in soybean, where extended linkage disequilibrium can reduce gene-level resolution of GWAS signals [40,41]. Integration of genetic mapping with functional annotation and transcriptomic data can therefore provide complementary evidence to prioritize biologically plausible genes within associated genomic regions [13,33]. In the present study, we combined genomic position, functional annotation, and tissue-specific expression to prioritize candidate genes within the high-confidence loci. This approach prioritized 112 candidate genes across 18 of the 21 loci (Figure 7 and Table S5).

The prioritized genes represented diverse biological processes, with the largest groups associated with hormone regulation, cell-wall remodeling, primary metabolism, stress and phenology, gene regulation, water and nutrient transport, redox and energy metabolism, and plant growth and development. Additional candidates were related to protein folding and quality control, protein turnover, photosynthesis, and seed development and storage. This broad functional diversity is consistent with the complex genetic architecture of soybean agronomic traits, in which phenotypic variation can reflect the combined contributions of multiple loci and interconnected developmental and metabolic processes [3].

Tissue-expression profiling provided an additional layer of information for prioritizing candidate genes. Genes within the identified loci showed contrasting expression patterns across vegetative and reproductive tissues, including leaves, stems, roots, flowers, pods, seeds, and shoot meristems (Figure 6D and Table S4). Extensive tissue- and developmental-stage-specific transcription has previously been demonstrated in soybean transcriptome atlases, revealing substantial differences in gene expression patterns between vegetative and reproductive organs [31,32]. Accordingly, expression in tissues relevant to plant development or reproductive growth provides useful complementary information for distinguishing biologically plausible candidates within broad genomic intervals. Importantly, tissue-specific expression was used here as a prioritization criterion rather than as evidence of causality. High transcript abundance in a particular tissue can strengthen the biological plausibility of a positional candidate but does not demonstrate that allelic variation in that gene underlies the corresponding GWAS association.

The *q.VER8.15-1* interval illustrates this limitation particularly well. Five genes within this locus showed pronounced but distinct tissue-expression maxima, including SoyZH13_15G055500 in Leaf1, SoyZH13_15G025700 in Pod1, SoyZH13_15G025800 in Flower1, SoyZH13_15G031500 in Leafbud1, and SoyZH13_15G074800 in the shoot meristem (Table S4). Rather than identifying a single causal gene, these expression patterns indicate that the interval contains five biologically plausible candidates active in different tissues and developmental contexts [33,41,42]. Thus, the present data do not allow causal assignment to an individual gene within this locus. Similar integrative approaches in soybean have demonstrated that combining mapping signals with transcriptomic information can substantially narrow candidate-gene sets, whereas additional genetic or functional evidence is required to establish causality [33,41,43].

Overall, the convergence of cross-model GWAS support, genomic position, functional annotation, and tissue-expression information provides a focused set of candidate genes for subsequent investigation. Nevertheless, these complementary lines of evidence should be regarded as prioritization criteria rather than functional validation [40,41]. Fine-mapping, haplotype analysis, genotype-dependent expression profiling, and functional assays will be required to distinguish causal genes from linked candidates and determine the molecular mechanisms underlying the identified genomic associations [40,41].

The 21 high-confidence stable loci identified in this study represent potential genomic resources for soybean improvement, with the three presumably novel loci providing additional targets for further investigation. However, their application in marker-assisted breeding will require validation in independent populations and environments, followed by fine mapping and characterization of favorable alleles [39,44]. Integration of validated loci with genomic prediction may further facilitate the improvement of complex agronomic traits under variable growing conditions [45,46].

## 4. Materials and Methods

### 4.1. Plant Materials and Experimental Design

**Figure 8:**
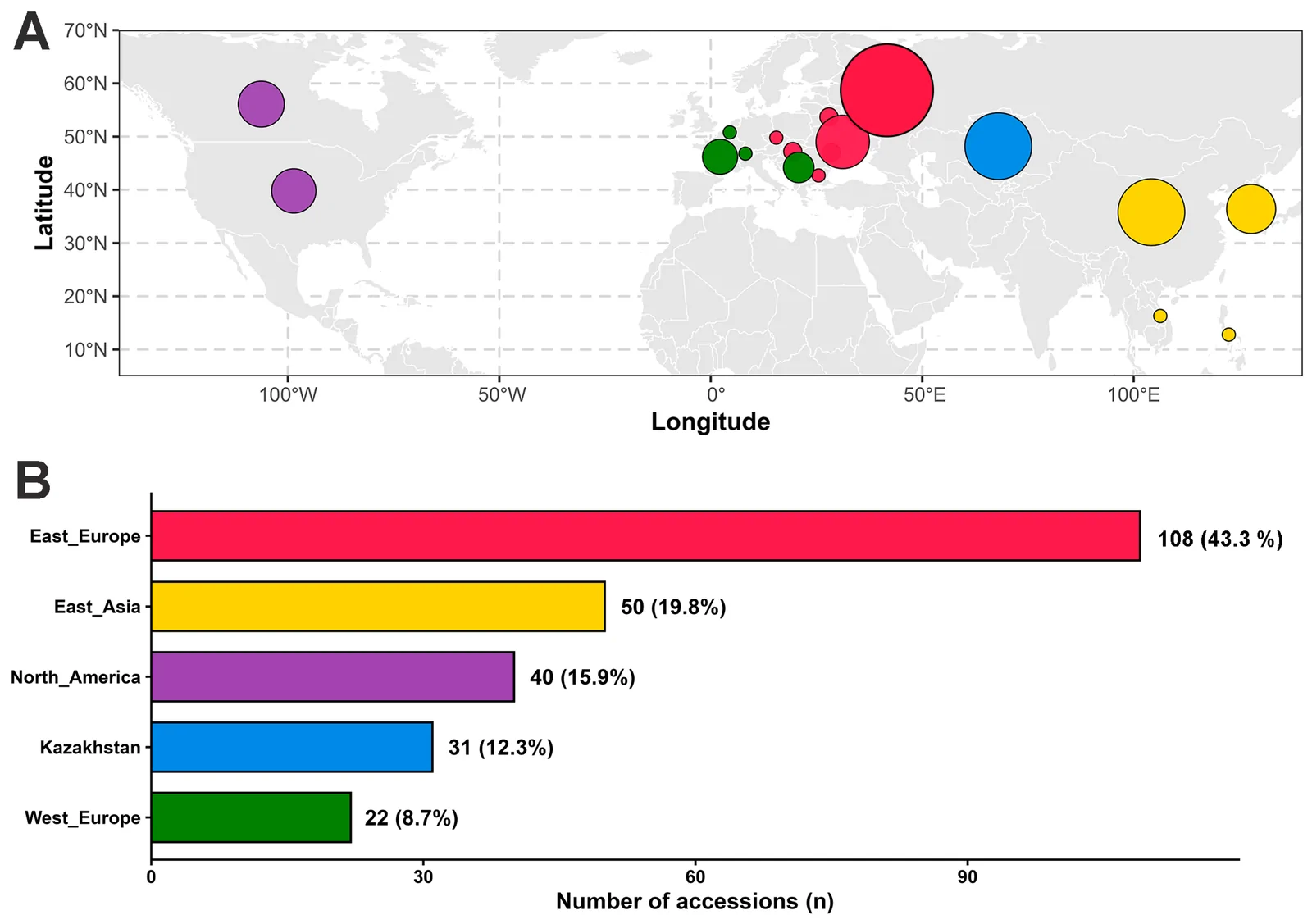
Geographic composition of the soybean association panel. (**A**) Distribution of accessions by country of origin; circle size is proportional to the number of accessions from each country. (**B**) Distribution of accessions among regions of origin.

A soybean panel comprising 252 accessions was used in the present study to investigate the genetic architecture of six major agronomic traits. The panel represented broad geographic diversity, encompassing cultivars and breeding lines from 20 countries across Europe, Asia, and North America (Figure 8 and Table S6).

Field experiments were conducted over six consecutive growing seasons (from 2018 to 2023) at the experimental fields of the Kazakh Research Institute of Agriculture and Plant Growing (KRIAPG) located in southeastern Kazakhstan (43°14′03″ N and 76°42′00″ E). This region represents the principal soybean-growing area of Kazakhstan and is characterized by a sharply continental climate with pronounced interannual variability in temperature, precipitation, and growing-season length (Figure S2) [2,28]. The present experiment therefore represents a multi-season, single-location design, in which interannual climatic variation provides a framework for evaluating the consistency of genetic effects across growing seasons at the experimental site.

The field trial was established independently in each growing season using a randomized complete block design (RCBD) with two field replicates, with each replicate representing a complete randomized block containing all evaluated accessions. Each accession was planted in a two-row plot, with each row measuring 1.0 m in length and containing 25 seeds, resulting in 50 plants per plot under optimal field emergence conditions. The order of accessions within each replicate was randomized annually to minimize positional bias and environmental heterogeneity across the experimental field. All field management practices, including land preparation, sowing, weed control, irrigation (five times between the flowering and pod-filling stages), and plant protection, followed standard agronomic recommendations [2,28] adopted for soybean cultivation at KRIAPG and were applied uniformly to all accessions throughout the six experimental years.

### 4.2. Phenotypic Data Analysis

Phenotypic evaluation focused on six key traits: two phenological traits (VER2; VER8), one morphological trait (PH), and three yield-related traits (NSP; YP; TSW). Soybean developmental stages were determined according to the growth stage classification system of Fehr and Caviness (1977) [47]. The same experimental protocol and field management practices were applied throughout the six growing seasons to minimize non-genetic sources of variation.

Phenotypic data collected during each growing season were subjected to statistical analyses prior to GWAS to characterize phenotypic variation and obtain season-adjusted phenotypic values. Descriptive statistics, including the minimum, maximum, mean, standard deviation (SD), CV, and frequency distribution, were calculated separately for each growing season and for the combined dataset.

The phenotypic dataset was partially unbalanced because observations were unavailable for genotype × growing season × replicate combinations. Missing observations occurred primarily when individual accessions did not reach physiological maturity within a particular growing season, preventing reliable recording of the corresponding maturity- and/or harvest-related traits. These observations were recorded as NA and were not imputed. When data were unavailable in one field replicate but available in the other, the available observation was retained for statistical analysis. No phenotypic observations were removed based on statistical outlier detection. Consequently, the number of observations available for analysis differed among traits, resulting in the trait-specific degrees of freedom reported in Table 1.

The effects of genotype, growing season, and genotype × growing season interaction were evaluated using analysis of variance (ANOVA). The broad-sense heritability (*H*^2^) across growing seasons was calculated from the estimated variance components according to the standard procedure for multi-season experiments [48,49].

To obtain genotype-specific adjusted means across the six growing seasons while accounting for environmental variation, BLUEs were calculated using linear mixed models [22,23]. Because the primary objective was to obtain genotype-specific phenotypic values for subsequent GWAS, accessions were treated as fixed effects, whereas growing seasons and genotype × growing season interactions were modeled as random effects. Consequently, BLUEs rather than BLUPs were estimated. The resulting BLUE values were used as the common phenotypic dataset for all three subsequent GWAS approaches. This across-season approach was intended to prioritize genomic associations with season-adjusted genotype performance rather than associations specific to individual growing seasons. Accordingly, growing season-specific marker effects potentially arising from genotype × growing season interactions were not the primary target of the BLUE-based GWAS.

Relationships among agronomic traits were evaluated using Pearson’s correlation coefficients based on the BLUE values. Correlation coefficients were visualized as a heatmap. All statistical analyses were performed in R v4.5.2, packages *lme4*, *emmeans*, *lmerTest*, *broom.mixed*, *ggcorrplot*, *ggraph*, *Hmisc*, *igraph*, *janitor*, *naniar*, *patchwork*, *performance*, *rnaturalearth*, *rnaturalearthdata*, *sf*, *skimr* and *tidygraph* [50].

### 4.3. Whole-Genome Resequencing and SNP Discovery

Whole-genome resequencing data were processed using a variant-discovery pipeline adapted from Lu et al. (2020) [51]. High-quality paired-end reads were mapped to the soybean reference genome *Glycine max* cv. Zhonghuang 13 (ZH13 v2.0) using BWA-MEM with default alignment parameters [52]. Sequence Alignment Map (SAM) files were converted to binary format (BAM) and coordinate-sorted using SAMtools v1.9 [53]. PCR duplicates were identified and marked using Picard tools v2.18.29 (MarkDuplicates) [54].

Genotype calling was performed across the panel using the Genome Analysis Toolkit (GATK) framework v4.1.2.0 [55]. Raw SNPs and InDels were called for individual accessions using HaplotypeCaller in GVCF mode and subsequently combined using GenotypeGVCFs for joint variant calling [56]. Strict quality control was executed via GATK VariantFiltration (QD < 2.0, FS > 60.0, SOR > 3.0, MQ < 40.0) and PLINK (v1.90) [57]. Only high-confidence SNPs with minor allele frequency (MAF) ≥ 0.05, missing genotype rate ≤ 10%, and mean depth ≥ 4x were retained (resulting in a final dataset of 2,019,772 SNPs) for downstream population structure and association analyses.

### 4.4. Population Genetic Analyses

Genome-wide LD was calculated using PLINK v1.90 as the squared correlation coefficient (*r*^2^) between pairs of intrachromosomal SNPs [57]. Marker pairs separated by ≤5 Mb were retained, and pairwise *r*^2^ values were summarized in 25 kb distance bins [12]. LD decay was determined from the five-point moving-average smoothed mean *r*^2^ curve using *r*^2^ = 0.20 as the threshold, with the decay distance estimated by linear interpolation between adjacent bins. Chromosome-specific LD decay was additionally calculated to account for variation in LD across the genome. The genome-wide LD decay distance was subsequently used to define LD intervals for locus consolidation and candidate gene identification.

Population structure was evaluated by principal component analysis (PCA) using GAPIT version 3 [15], and the genomic kinship matrix was estimated using the VanRaden algorithm implemented in the same package [58]. Population structure was controlled for by including the first three principal components (PC1–PC3) and the genomic kinship matrix as covariates in all GWAS.

### 4.5. Genome-Wide Association Analysis

GWAS was performed using genotype-specific BLUE values calculated across six growing seasons. The same BLUE dataset was analyzed using three complementary multi-locus GWAS approaches, IIIVmrMLM, MLMM, and BLINK, to identify genomic regions associated with the six agronomic traits. In the present study, IIIVmrMLM was applied to the across-season BLUE phenotypes to detect main effects of QTN. Although the IIIVmrMLM framework can explicitly model QTN-by-environment interactions when environment-specific phenotypic observations are provided [21], such interactions were not directly estimated in the present BLUE-based GWAS. MLMM and BLINK were used as complementary association frameworks [19,20].

An initial screening threshold of *p* < 1.0 × 10^−4^ was applied to retain potentially informative marker–trait associations for subsequent LD-based locus consolidation and cross-model validation. This threshold was used exclusively for initial association screening and should not be interpreted as a Bonferroni-corrected genome-wide significance threshold. Based on the 2,019,772 SNPs included in the GWAS, the Bonferroni-corrected threshold was *p* < 2.48 × 10^−8^ (0.05/2,019,772). Final locus prioritization was based on LD-defined consolidation followed by concordant support across the three GWAS frameworks, as described below.

### 4.6. Identification of High-Confidence Genomic Loci

Significant QTNs identified by the three multi-locus GWAS models (IIIVmrMLM, BLINK, and MLMM) were first consolidated into genomic loci according to their physical positions and local LD structure [3,12]. Significant SNPs located within the genome-wide LD decay interval (*r*^2^ = 0.20; 2.196 Mb) were considered to represent the same genomic locus, and the SNP with the strongest statistical support was designated as the lead SNP.

The robustness of the identified loci was subsequently evaluated by cross-model comparison among IIIVmrMLM, BLINK, and MLMM. Loci supported by at least two of the three GWAS models were considered cross-model-validated, yielding a set of 80 loci. A more stringent criterion was then applied to identify genomic regions supported by all three GWAS models. This procedure retained 21 loci, designated as high-confidence stable loci, for subsequent comparison with previously reported soybean QTLs and candidate-gene analyses. Thus, in the present study, the term ‘high-confidence stable locus’ refers to a genomic region identified from the six-season phenotypic dataset and consistently supported by IIIVmrMLM, BLINK, and MLMM.

To compare the 21 high-confidence stable loci with previously reported soybean QTLs, physical coordinates originally anchored to the Zhonghuang 13 (ZH13 v2.0) reference genome were converted to Williams 82 (Wm82.a2.v1) coordinates using SoyOmics [59]. The converted genomic intervals were subsequently compared with previously reported soybean QTLs available in SoyBase [30]. A locus was considered to overlap a previously reported QTL when its converted Wm82.a2.v1 interval physically intersected the corresponding SoyBase QTL interval. Loci showing neither positional overlap with previously reported SoyBase QTLs nor co-localization with established soybean genes were classified as presumably novel.

Thus, in the present study, the term ‘high-confidence stable locus’ is used operationally to denote a genomic region identified from genotype-specific BLUEs estimated across six growing seasons and consistently supported by IIIVmrMLM, BLINK, and MLMM after LD-based locus consolidation. This designation refers to cross-model robustness of associations with across-season genotype performance and does not imply that the corresponding marker effect was independently significant in each growing season or necessarily exceeded the Bonferroni-corrected genome-wide significance threshold.

### 4.7. Candidate Gene Selection and Functional Annotation

Candidate genes within the high-confidence genomic loci were retrieved from the Zhonghuang 13 (ZH13 v2.0) genome annotation. Descriptions of candidate genes were retrieved from SoyOD [60]. Functional annotation was performed using SoyMD [61] database. Candidates were prioritized based on genomic position, annotated biological function relevant to the traits under investigation, and tissue-expression evidence.

GO annotations were classified by Biological Process, Molecular Function, and Cellular Component, and then summarized into broader functional categories for visualization. Because individual genes could be assigned to multiple categories, the reported counts represent gene–category assignments rather than unique genes.

Tissue-specific expression was evaluated using the SoyMD [61] transcriptome dataset. For candidate-gene prioritization in the present study, genes with transcript abundance ≥ 200 TPM in at least one tissue were operationally classified as highly expressed. Genomic position, functional annotation, and expression evidence were integrated to obtain the final set of prioritized candidate genes. Genomic position, functional annotation, and expression evidence were integrated to obtain the final set of prioritized candidate genes. These criteria were used exclusively for candidate prioritization and were not considered evidence of functional validation or causality.

## 5. Conclusions

Six consecutive years of field evaluation combined with whole-genome resequencing and complementary GWAS models identified 21 high-confidence genomic loci associated with soybean phenology, plant architecture, and yield components. Ten loci co-localized with regions containing established soybean regulators, including *E2*, *E3*, *GmDt2*, and *POWR1*, support the biological plausibility of the association results. Three loci (*q.VER2.13-1*, *q.YP.01-1*, and *q.TSW.15-1*) showed no positional overlap with genes and QTLs included in the SoyBase comparison and were therefore classified as presumably novel. Integration of genomic position, functional annotation, and tissue-expression evidence further prioritized 112 candidate genes across 18 loci. The three presumably novel loci and their associated candidate genes represent priority targets for independent validation, fine-mapping, haplotype analysis, and functional characterization. Importantly, because the present study was conducted at a single location, validation across additional soybean populations and geographically distinct environments will be required to determine the broader environmental stability and breeding utility of these loci.

## Figures and Tables

**Table 1:** ANOVA and *H*^2^ for phenological and agronomic traits across genotypes and growing seasons.

Factors	Df	SS	MS	*F*-Value	*p*-Value	*H* ^2^
**Flowering time (VER2, days)**						
Genotype	251	198,744.1	791.809	341.822	<2 × 10^−16^	0.7425
Growing season	5	19,102.83	3820.565	1649.330	<2 × 10^−16^	
Genotype × Growing season	1252	46,322.32	36.999	15.972	<2 × 10^−16^	
Residuals	1509	3495.50	2.316			
**Maturity time (VER8, days)**						
Genotype	251	962,897.8	3836.246	427.976	<2 × 10^−16^	0.9129
Growing season	5	9047.775	1809.555	201.876	<2 × 10^−16^	
Genotype × Growing season	1243	69,579.34	55.977	6.245	<2 × 10^−16^	
Residuals	1474	13,212.50	8.964			
**Plant height (PH, cm)**						
Genotype	251	2,022,832	8059.093	100.704	<2 × 10^−16^	0.7579
Growing season	5	272,632.4	54,526.480	681.347	<2 × 10^−16^	
Genotype × Growing season	1237	257,767.6	208.381	2.604	<2 × 10^−16^	
Residuals	1446	115,719.7	80.027			
**Number of seeds per plant (NSP, count)**						
Genotype	251	587,287.5	2339.791	14.641	<2 × 10^−16^	0.3477
Growing season	5	299,469.3	59,893.870	374.790	<2 × 10^−16^	
Genotype × Growing season	1237	571,291.2	461.836	2.890	<2 × 10^−16^	
Residuals	1446	231,080.4	159.807			
**Seed yield per plant (YP, g)**						
Genotype	251	79,066.43	315.006	22.203	<2 × 10^−16^	0.3132
Growing season	5	52,802.59	10,560.520	744.364	<2 × 10^−16^	
Genotype × Growing season	1240	99,863.68	80.535	5.677	<2 × 10^−16^	
Residuals	1458	20,685.09	14.187			
**Thousand-seed weight (TSW, g)**						
Genotype	251	2,705,500	10,778.88	88.217	<2 × 10^−16^	0.2350
Growing season	5	170,375.6	34,075.120	278.879	<2 × 10^−16^	
Genotype × Growing season	1240	8,458,913	6821.704	55.830	<2 × 10^−16^	
Residuals	1458	178,147.3	122.186			

Notes: Df—degree of freedom; SS—sum of square; MS—mean of square; *H*^2^—broad-sense heritability. Differences in degrees of freedom among traits reflect missing phenotypic observations and the resulting partially unbalanced dataset.

**Table 2:** High-confidence stable marker–trait associations for six soybean agronomic traits validated across three GWAS models.

Lead SNP	Chromosome	Interval	QTL	*p*–Value	*PVE* (%)	GWAS Model with Highest Significance
S06_18429048	6	15,878,636–18,449,510	*q.VER2.06-1*	5.22 × 10^−38^	5.99	IIIVmrMLM
S10_45267444	10	44,983,103–45,552,240	*q.VER2.10-1*	7.69 × 10^−23^	2.14	IIIVmrMLM
S13_28073418	13	28,073,418–28,121,434	*q.VER2.13-1*	9.33 × 10^−21^	1.60	IIIVmrMLM
S18_7723643	18	7,624,758–9,322,836	*q.VER2.18-1*	2.27 × 10^−10^	0.81	IIIVmrMLM
S19_46965390	19	45,001,588–48,047,243	*q.VER2.19-1*	7.63 × 10^−28^	5.10	IIIVmrMLM
S11_720016	11	22,022–3,711,685	*q.VER8.11-1*	9.99 × 10^−29^	3.00	IIIVmrMLM
S15_5260173	15	2,044,697–7,546,198	*q.VER8.15-1*	3.75 × 10^−22^	1.00	IIIVmrMLM
S19_47287186	19	47,172,329–48,256,419	*q.VER8.19-1*	5.91 × 10^−26^	2.66	IIIVmrMLM
S10_45257173	10	45,041,135–45,552,240	*q.PH.10-1*	2.21 × 10^−15^	1.35	IIIVmrMLM
S18_55969461	18	54,039,880–56,151,405	*q.PH.18-1*	1.96 × 10^−18^	2.51	IIIVmrMLM
S19_45487455	19	44,591,591–45,977,296	*q.PH.19-1*	3.51× 10^−24^	4.85	IIIVmrMLM
S19_48204814	19	48,039,689–48,348,069	*q.PH.19-2*	5.99 × 10^−27^	5.99	IIIVmrMLM
S20_6620602	20	3,074,803–24,303,023	*q.PH.20-1*	3.92 × 10^−18^	2.52	IIIVmrMLM
S09_1699098	9	1,129,550–1,785,273	*q.NSP.09-1*	1.29 × 10^−6^	1.45	IIIVmrMLM
S10_45323146	10	44,602,366–45,523,711	*q.NSP.10-1*	2.42 × 10^−9^	1.01	IIIVmrMLM
S19_44766594	19	44,766,594–44,805,796	*q.NSP.19-1*	5.40 × 10^−16^	4.36	IIIVmrMLM
S20_20307466	20	8,357,455–33,083,463	*q.NSP.20-1*	8.80 × 10^−9^	1.06	IIIVmrMLM
S01_52792832	1	52,578,162–52,792,832	*q.YP.01-1*	9.70 × 10^−9^	2.13	IIIVmrMLM
S20_7229020	20	3,426,776–33,040,783	*q.YP.20-1*	7.37 × 10^−17^	5.34	IIIVmrMLM
S15_44011324	15	43,727,202–44,083,921	*q.TSW.15-1*	1.09 × 10^−7^	5.53	IIIVmrMLM
S19_37069082	19	37,069,082–40,569,132	*q.TSW.19-1*	3.55 × 10^−6^	18.12	BLINK

Note: *PVE*—phenotypic variance explained by the lead SNP.

**Table 3:** Positional comparison of the 21 high-confidence stable loci with previously reported soybean genes and QTLs in SoyBase.

QTL	Region Based on Wm82.a2.v1	No. of Overlapping SoyBase QTLs	Positional Known Genes	Relevant Previously Reported Traits
*q.VER2.06-1*	Gm06: 15,865,644–18,619,504	26	–	First flower; reproductive period; R8 full maturity
*q.VER2.10-1*	Gm10: 42,445,833–43,021,267	2	*E2* (*GmGIa*)	Seed Leu; seed Ser
*q.VER2.13-1*	Gm13: 26,896,765–26,945,435	0	–	–
*q.VER2.18-1*	Gm18: 7,623,678–9,733,932	9	–	Leaf carotenoid content
*q.VER2.19-1*	Gm19: 43,639,997–46,534,068	66	*E3* (*GmPhyA3*)	First flower; reproductive period; R8 full maturity
*q.VER8.11-1*	Gm11: 11–3,688,887	18	–	First flower; R8 full maturity; seed yield
*q.VER8.15-1*	Gm15: 1,968,118–7,495,737	46	–	Plant height; pod number; seeds per plant
*q.VER8.19-1*	Gm19: 45,685,540–46,743,147	11	–	First flower; pod number; seed set
*q.PH.10-1*	Gm10: 42,503,954–43,021,267	2	*E2* (*GmGIa*)	Seed Leu; seed Ser
*q.PH.18-1*	Gm18: 51,480,260–53,533,524	14	*GmDt2*	First flower; seed set
*q.PH.19-1*	Gm19: 43,230,278–44,571,804	8	*E3* (*GmPhyA3*)	Plant height; node number; stem termination type
*q.PH.19-2*	Gm19: 46,526,514–46,839,610	4	–	Canopy width; interbranch length
*q.PH.20-1*	Gm20: 3,032,414–20,660,856	11	–	First flower; R8 full maturity
*q.NSP.09-1*	Gm09: 1,132,934–1,779,330	1	–	SDS
*q.NSP.10-1*	Gm10: 42,055,769–42,992,799	3	*E2* (*GmGIa*)	Soybean mosaic virus; seed Leu; seed Ser
*q.NSP.19-1*	Gm19: 43,405,296–43,444,145	0	*E3* (*GmPhyA3*)	–
*q.NSP.20-1*	Gm20: 8,235,449–28,849,353	22	*POWR1/GmCCT*	Seed weight; first flower; reproductive period; R8 full maturity
*q.YP.01-1*	Gm01: 50,115,093–50,331,118	0	–	–
*q.YP.20-1*	Gm20: 3,379,584–28,797,537	26	*POWR1/GmCCT*	Seed weight; seed protein; seed oil
*q.TSW.15-1*	Gm15: 41,557,833–41,921,123	0	–	–
*q.TSW.19-1*	Gm19: 35,679,067–39,203,967	9	*E3* (*GmPhyA3*)	Seed oleic; seed Gly

## Data Availability

The original contributions presented in this study are included in the article/Supplementary Materials. The raw resequencing data are available at https://zenodo.org/records/15392469, accessed on 20 April 2026 (DOI 10.5281/zenodo.15392468). Further inquiries can be directed to the corresponding author(s).

## Supplementary Materials

The following supporting information can be downloaded at https://www.mdpi.com/article/10.3390/plants15182800/s1, Figure S1: Chromosome-specific linkage disequilibrium (LD) decay showing mean and median pairwise LD (*r*^2^) as a function of physical distance between SNPs; Figure S2: Meteorological conditions of the experimental site during the May-September growing seasons over six years (2018–2023); Table S1: Descriptive statistics of six agronomic traits evaluated across six consecutive growing seasons; Table S2: Summary of marker-trait associations (MTAs) and the stepwise identification of significant QTLs using three GWAS methods; Table S3: Detailed co-localization of QTL identified in this study with previously reported soybean QTL from the SoyBase database; Table S4: Expression profiles and functional annotation of candidate genes within the identified QTL regions; Table S5: Prioritized candidate genes within the identified genomic loci and their associated biological mechanisms; Table S6: List of soybean accessions used in this study and their geographic origins.

## Abbreviations

The following abbreviations are used in this manuscript:

ANOVA	Analysis of Variance
BLINK	Bayesian-Information and Linkage-Disequilibrium Iteratively Nested Keyway
BLUE	Best Linear Unbiased Estimators
GWAS	Genome-Wide Association Study
LD	Linkage Disequilibrium
MLMM	Multi-Locus Mixed Model
NSP	Number of Seeds per Plant
PH	Plant Height
QTL	Quantitative Trait Loci
SNP	Single Nucleotide Polymorphism
TSW	Thousand Seeds Weight
VER2	Flowering time
VER8	Maturity time
YP	Yield per Plant
